# Assessing the Health Impact of Trade: A Call for an Expanded Research Agenda: Comment on "The Trans-Pacific Partnership: Is It Everything We Feared for Health?"

**DOI:** 10.15171/ijhpm.2016.126

**Published:** 2016-09-14

**Authors:** Courtney McNamara

**Affiliations:** Department of Sociology and Political Science, Norwegian University of Science and Technology, Trondheim, Norway.

**Keywords:** Trade, Health, Social Policy, Labour Markets

## Abstract

Labonté et al provide an insightful analysis of the Trans-Pacific Partnership (TPP) and its impact on a selection of important health determinants. Their work confirms concerns raised by previous analyses of leaked drafts and offers governments serious and timely reasons to carefully consider provisions of the agreement prior to moving forward with ratification. It also contributes more generally to a growing literature focused on identifying the health impacts of trade. This commentary uses the authors’ analysis as a starting point to reflect on two interrelated issues relevant both for taking seriously one of the article’s main recommendations and future work in the area of trade and health.

Labonté et al^1^ provide an insightful analysis of the Trans-Pacific Partnership (TPP) and its impact on a selection of important health determinants. Their work confirms concerns raised by previous analyses of leaked drafts^2,3^ and offers governments serious and timely reasons to carefully consider provisions of the agreement prior to moving forward with ratification. It also contributes more generally to a growing literature focused on identifying the health impacts of trade. This commentary uses the authors’ analysis as a starting point to reflect on two interrelated issues relevant both for taking seriously one of the article’s main recommendations and future work in the area of trade and health.

Like previous analyses, Labonté et al find a number of potentially serious health risks associated with the TPP. These risks relate largely to access to medicines, health services, tobacco and alcohol control, and nutrition-related health. Their findings are arrived at using a method which is both well-established in other policy areas and specifically recommended for investigating the health impacts of global economic agreements: health impact analysis.^2,4^ The authors limit their analysis in number of ways, for example, by focusing on potential health risks (and not potential health benefits), by excluding certain Chapters of the agreement from their assessment and by limiting their attention to those risks identified by previous analyses of leaked TPP text. Sound reasons are presented for these methodological decisions which lead to one of their key recommendations: a call for more comprehensive assessments of the TPP.

Taking this recommendation seriously, however, requires the recognition that thus far, very little attention has been devoted to identifying potential health impacts of trade with reference to broader determinants of health.^5,6^ That is, the bulk of trade and health assessments (including, but not limited to, those of the TPP) have focused almost entirely on relatively ‘downstream’ determinants of health associated with biomedical factors (eg, access to medicines and health services) and lifestyle-focused/behavioral risk factors (eg, related to smoking, alcohol consumption, and diet). This is despite the fact that many of the most publicly contentious issues surrounding contemporary trade arrangements relate to factors considered to be fundamental determinants of health such as employment, income, and economic inequality.^7^

One implication of this gap in the literature is that more thorough assessments of the TPP cannot rely, as the authors did, on previously identified links but will need to draw on alternative frameworks for analysis. Existing analyses of some of the economic impacts of the TPP offer a starting point for thinking about these issues. One analysis for example, has found that the TPP will likely lead to losses in employment and increases in inequality in all participating countries.^8^ Necessary then are frameworks for evaluating whether and how such changes might impact health, particularly across such an economically, socially, and politically diverse set of countries. Employment loss for instance, is likely to be more health damaging in countries with less generous unemployment protection.^9^

Relatedly, taking seriously the authors’ call for more comprehensive health assessments requires further consideration of the tools which are best suited to assess the health impacts of trade. Within trade and health literature, there has been little methodological reflection on what theories, research designs or methods best inform analyses of trade and health. While health impact assessment has proved useful in a small but growing number of trade policy investigations, this method is not without its limitations.^10,11^ Other assessments of the health impacts of trade have drawn on a variety of both quantitative^12,13^ and qualitative methods,^6,14^ but questions remain. For example, what are the broader methodological lessons that can be drawn from the utilization of these different approaches, what are the different theories which currently underpin trade and health analyses, and what other methods and theoretical frameworks might prove useful at the nexus of trade and health?

The broader area of health policy analysis offers potentially valuable analytical frameworks for trade and health assessments.^15^ The health policy triangle, for example, draws particular attention to the interaction of contextual factors, processes and actors that should be systematically considered in analyzing the content of policy.^15,16^ In considering the health impacts of the TPP, these concepts can bring respective attention to the fact that: (1) The TPP will produce varying health impacts depending on the different socio-political contexts of countries, (2) The potential health impacts of the TPP may depend on whether provisions are phased in, allow for revisions, or are subject to public consideration or enforcement procedures, and (3) The distribution of power among TPP stakeholders can also affect health. TPP stakeholders include consumers, workers, multinational corporations, national governments, state and international institutions, and the public at large. A distribution of power favorable to multinational firms for example, may mean that laws and regulations with positive implications for health (but negative implications for private profit) are delayed, weakened, or overturned by corporate actors.^17^

With these gaps in the literature and methodological questions in mind, what is needed is a more focused and strategic research agenda for assessing both the health impacts of the TPP and trade more generally. Because the area of trade and health is concerned with issues that are relevant for an array of disciplines (from public health to social policy, sociology, and political science), establishing a comprehensive research agenda will require the integration of public health specialists, epidemiologists, sociologists, social policy experts, lawyers, political scientists, and economists into trade and health research groups. Current acknowledgements of the links between trade and health call for the incorporation of health impact assessments within international agreements, strengthened representation of public health within economic negotiations and greater coherence between trade and health policy.^4,18,19^ Expanding our understanding, both of the links between trade and health and of the most effective tools for assessing these links, not only strengthens these calls, but is crucial to successfully responding to them.

## Ethical issues

Not applicable.

## Competing interests

Author declares that she has no competing interests.

## Author’s contribution

CM is the single author of the paper.
